# Dr. Mott

**Published:** 1850-11

**Authors:** 


*Dr. Mott.*—Dr. Mott has returned from Europe, and accepted the appointment of Emeritus Professor of Surgery in the College of Physicians and Surgeons, in the city of New York. It is expected that he will lecture occasionally at that Institution during the lecture session now in progress.

